# Deciphering protein long-chain *S*-acylation using mass spectrometry proteomics strategies

**DOI:** 10.1039/d5cb00146c

**Published:** 2025-09-08

**Authors:** Anneroos E. Nederstigt, Samiksha Sardana, Marc P. Baggelaar

**Affiliations:** a Biomolecular Mass Spectrometry and Proteomics, Bijvoet Center for Biomolecular Research and Utrecht Institute for Pharmaceutical Sciences, University of Utrecht, Padualaan 8 Utrecht 3584 CH The Netherlands m.p.baggelaar@uu.nl; b Netherlands Proteomics Center, Padualaan 8 Utrecht 3584 CH The Netherlands

## Abstract

Protein long-chain *S*-acylation, the reversible attachment of fatty acids such as palmitate to cysteine residues *via* thioester bonds, is a widespread post-translational modification that plays a crucial role in regulating protein localization, trafficking, and stability. Despite its prevalence and biological relevance, the study of long-chain *S*-acylation has long lagged behind that of other dynamic PTMs due to the hydrophobic nature and lability of the lipid modification, which complicate conventional proteomic workflows. Recent advances in mass spectrometry-based strategies have significantly expanded the toolbox for studying long-chain *S*-acylation, with improved workflows enabling more sensitive, site-specific, and quantitative analysis. This review summarizes key developments from the past decade across both direct and indirect mass spectrometry-based strategies, including acyl-biotin exchange, lipid metabolic labeling, and novel enrichment and fragmentation methods. We also highlight emerging challenges in distinguishing lipid-specific modifications, achieving robust quantification, and mitigating artifacts from *in vitro* systems, while outlining future directions to advance functional and therapeutic exploration of the *S*-acyl-(prote)ome.

## Abstract 2

Deciphering protein long-chain *S*-acylation using mass spectrometry proteomics strategies.

## Graphical abstract

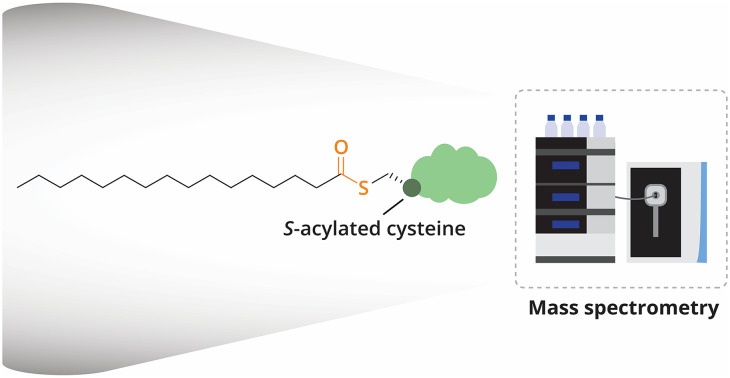

## 1. Introduction

Protein long-chain *S*-acylation involves the attachment of C14–C20 fatty acids to proteins at cysteine residues through a thioester bond. This post-translational modification (PTM) is often referred to as *S*-palmitoylation, with palmitate (C16 : 0) being the most common lipid attached. Long-chain *S*-acylation is among the most widespread PTMs in mammals, affecting approximately 25% of the human proteome,^1^ and it plays a critical role in regulating protein trafficking,^2–4^ protein–protein interactions,^5^ and protein stability.^6–8^

**Fig. 1:**
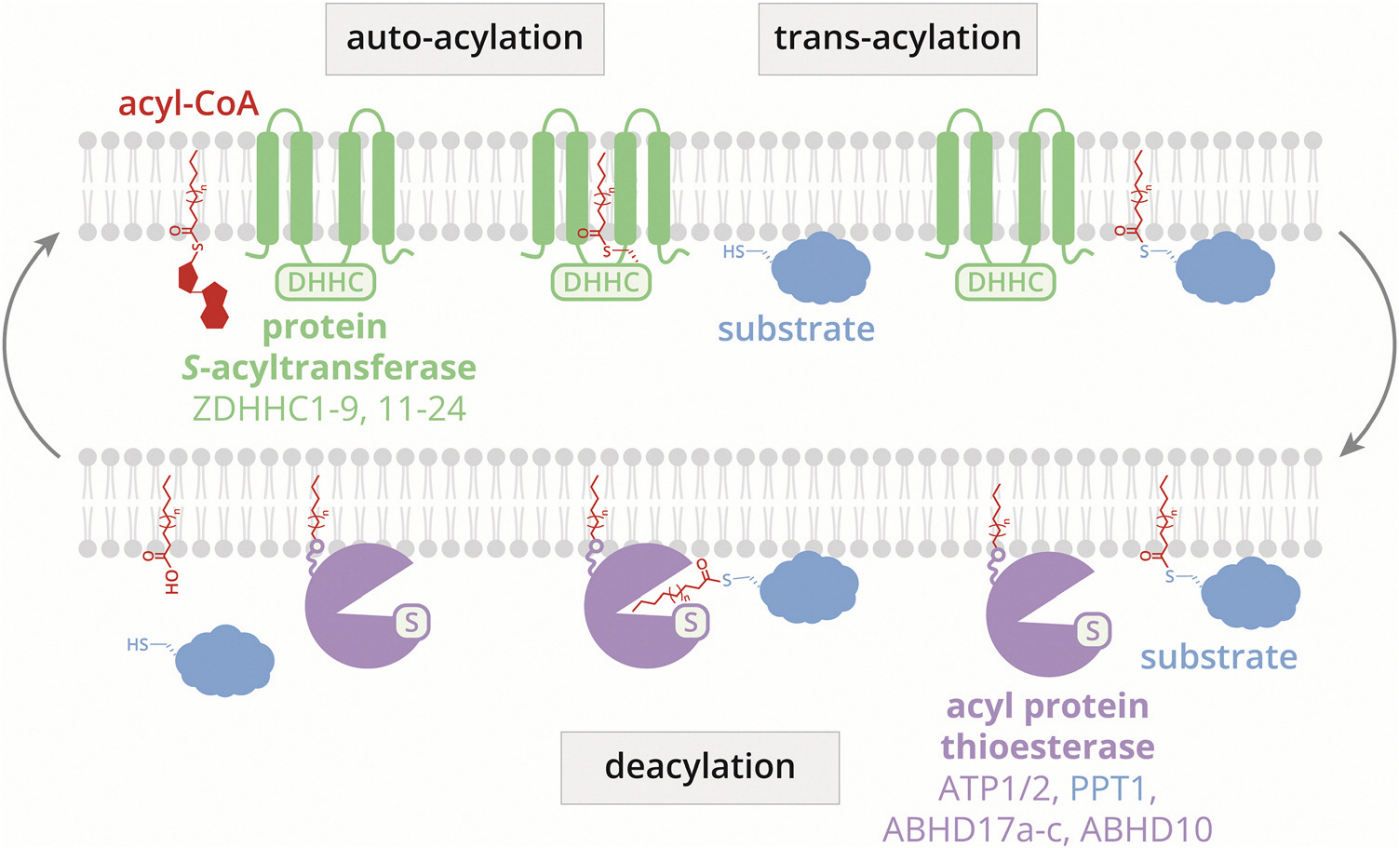
Overview of the *S*-acylation regulatory machinery. Long-chain *S*-acylation is regulated by 23 protein *S*-acyl transferases and seven validated acyl-protein thioesterases.

In contrast to other lipid PTMs, such as *N*-myristoylation and *S*-prenylation, long-chain *S*-acylation is unique in its reversible nature. The lipid modification is attached by the ZDHHC *S*-acyltransferase family, which includes 23 known human ZDHHCs (Fig. 1). Long-chain *S*-acylation sites in proteins do not exhibit a clear consensus sequence, although they often occur proximal to the membrane and are mostly surface-exposed.^9,10^ Substrate recruitment by ZDHHCs may involve co-localization through protein interactions, membrane-associated domains, or prior lipid PTMs.^11–15^

Deacylation, mediated by a growing group of acyl-protein thioesterases, creates a dynamic *S*-acylation cycle that is implicated in signalling cascades (Fig. 1). Of note, palmitoyl protein thioesterase 1 (PPT1) is a lysosomal palmitoyl protein thioesterase and may therefore not be involved in dynamic *S*-acylation cycles. The long-chain *S*-acylation machinery holds great potential as a drug target due to its prevalence in mammals, its dysregulation in pathological conditions,^16–24^ and its tight enzymatic control.

Mass spectrometry-based proteomics is arguably the most powerful strategy for analyzing PTMs and has greatly enhanced our understanding of other widespread reversible PTMs, such as acetylation and phosphorylation. Importantly, insights into kinase and acetylase signaling have led to successful therapeutic strategies aimed at altering the PTM states of proteins, such as the use of kinase and HDAC inhibitors in cancer therapy. However, knowledge of the extent and function of long-chain *S*-acylation lags that of other widespread PTMs. This gap can be partly attributed to the chemical properties of the lipid modification. The lipids are attached to proteins through a labile thioester bond, and their presence significantly increases the hydrophobicity of the bound peptides and proteins, making them challenging to analyze using standard proteomics strategies typically employed for more polar PTMs.^25^

As a result, our understanding of the role and extent of long-chain *S*-acylation has predominantly relied on indirect chemical biology strategies that circumvent these challenges during analysis. These strategies include acyl-biotin exchange (ABE), acyl-resin-assisted capture (acyl-RAC), and lipid metabolic labeling with alkyne- or azide-tagged lipids. In parallel, significant progress has also been made in the direct analysis of protein long-chain *S*-acylation.

Against this backdrop, this review highlights the advances made over the past decade in mass spectrometry-based strategies to analyze long-chain *S*-acylation in mammalian systems. We discuss the advantages and limitations of both direct and indirect approaches and offer perspectives on how emerging tools may further expand our ability to map, quantify, and functionally characterize this dynamic lipid modification.

## 2. Indirect analysis of protein long-chain *S*-acylation

Proteome-scale analysis of protein long-chain *S*-acylation through indirect strategies relies on the enrichment of long-chain *S*-acylated proteins and/or peptides. These enrichment strategies can be classified in cysteine-centric methods termed acyl-biotin exchange, and lipid-centric methods that rely on metabolic labelling with fatty acid analogues followed by click chemistry. In the next section we will discuss the progress made with these indirect strategies for proteome-wide analysis of long-chain *S*-acylation.

### 2.1 Acyl-biotin exchange and acyl-RAC

**Fig. 2:**
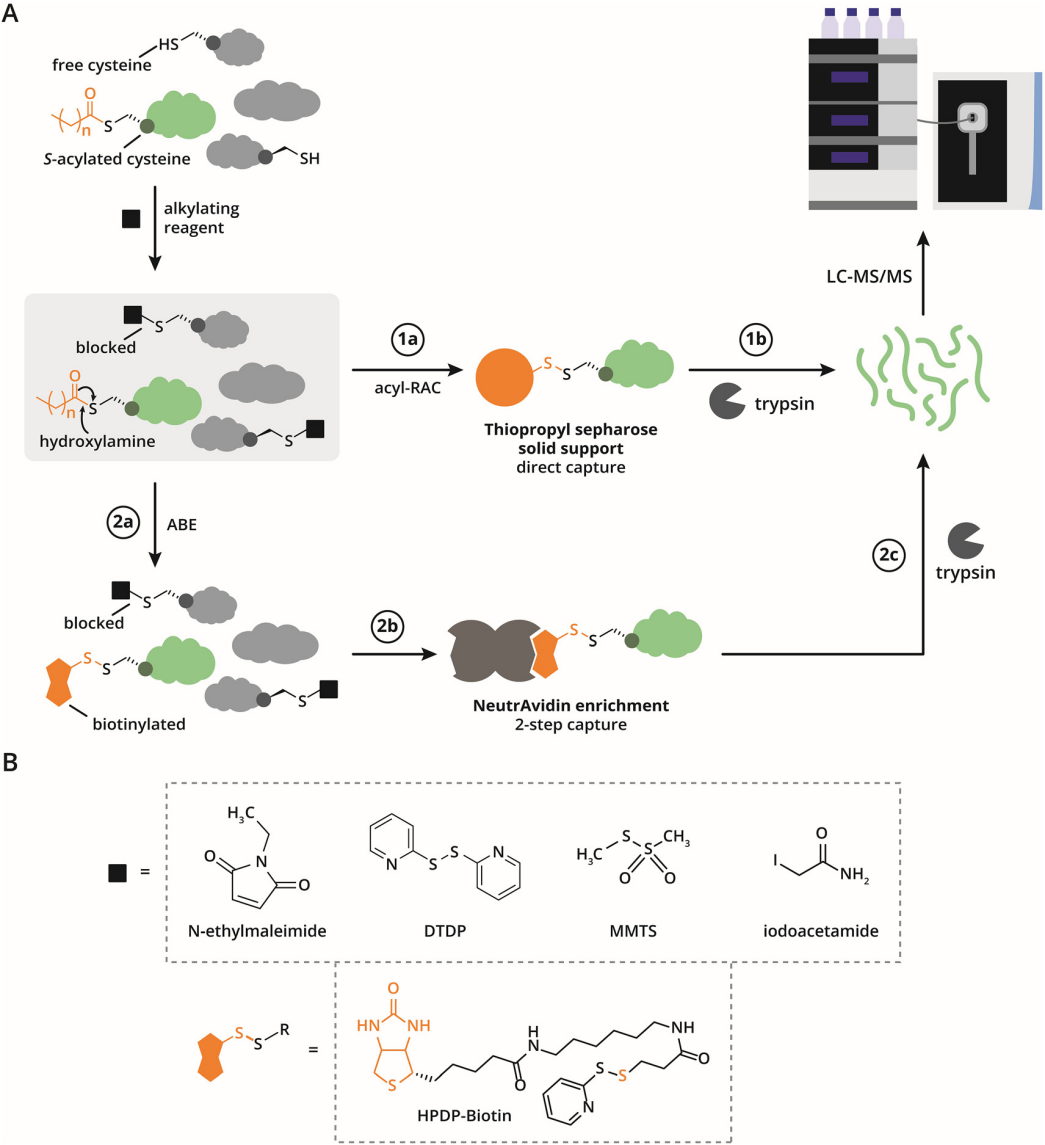
Acyl-biotin exchange and acyl-RAC workflows for identifying *S*-acylated proteins and modification sites *via* mass spectrometry. (A) Overview of ABE and acyl-RAC common to both workflows: proteins are treated with a reducing agent to reduce disulfide bonds into free thiols, while leaving *S*-acylated cysteines unaffected. Free thiols are then capped using an alkylating reagent. Acyl-RAC workflow: step 1a. Following hydroxylamine treatment, free thiols are captured on thiopropyl sepharose resin. Step 1b. Captured proteins are released from the resin with a reducing agent. Proteins are digested and measured by LC-MS/MS. ABE workflow: step 2a. Thioester bonds are selectively cleaved with hydroxylamine (neutral pH), converting *S*-acylated cysteines into free thiols, which are then biotinylated (*e.g.* using HPDP-biotin). Step 2b. Biotinylated proteins are enriched *via* NeutrAvidin resin and selectively eluted with a reducing agent. Step 2c. Eluted proteins are digested, cleaned, and analyzed using LC-MS/MS. In site-specific workflows proteins are digested prior to enrichment producing a mixture of biotinylated, alkylated and unmodified peptides (ssABE) or a mixture of thiopropyl sepharose captured peptides (site-specific acyl-RAC). (B) Chemical structures of commonly used cysteine-capping (alkylating) reagents, and the most widely used biotinylating reagent, HPDP-biotin.

The acyl-biotin exchange method enables the identification of *S*-acylated proteins or peptides through the enrichment of previously *S*-acylated cysteines. This approach was first described by Drisdel and Green in 2004.^26^ The key steps of the ABE strategy are: (1) blocking free cysteines, (2) hydrolyzing thioester-linked cysteines, and (3) selectively enriching the newly-exposed cysteines, which correspond to the previously *S*-acylated sites (Fig. 2A).

Roth *et al.* were the first to integrate the ABE method with mass spectrometry proteomics to identify long-chain *S*-acylated proteins.^27^ Their strategy involved blockage of free cysteines by *N*-ethylmaleimide (NEM), followed by hydrolysis of thioesters using hydroxylamine (HA) at a concentration of 1 M under neutral pH (7.4). The newly-exposed cysteines were then biotinylated with HPDP-biotin (Fig. 2A, step 2a, Fig. 2B). Enrichment of biotinylated proteins on streptavidin-conjugated solid supports, followed by mass spectrometry analysis, led to the identification of 47 putative long-chain *S*-acylated proteins in the yeast *Saccharomyces cerevisiae*. This pioneering study laid the groundwork for numerous ABE-related strategies designed to enhance sensitivity, streamline the protocol, and reduce false-positive identifications. These advancements have made ABE a pivotal technique for studying *S*-acylation and its biological significance.

#### 2.1.1 Enhanced sensitivity and reduction of false positives in ABE strategies through optimized cysteine capping

Efficient capping of free cysteines is a critical step in acyl-biotin exchange-related strategies to ensure sensitivity and prevent false positive identifications. Proper alkylation of free cysteines requires careful optimization of reaction conditions, particularly pH, to ensure efficient blocking of free thiols while preserving the integrity of thioester-linked *S*-acylation.

Cysteine capping is typically performed at pH 7.4 to balance reaction efficiency and selectivity. At lower pH, alkylation reagents react less effectively with thiols, while at higher pH, thioester bonds may hydrolyze, leading to loss of *S*-acylation and reduced specificity of the blocking reagent toward cysteines over other nucleophilic residues. To address these challenges, various alkylating agents have been employed (Fig. 2B). Among these, *N*-ethylmaleimide has been the most widely used due to its high reactivity with cysteine thiols. To further improve cysteine capping efficiency, some protocols have even implemented multiple sequential rounds of NEM treatment.^28^

Zhang *et al.* introduced methyl methanethiosulfonate (MMTS) as an alternative capping agent for ABE workflows, and it has since been successfully applied in several studies.^29–37^ Another commonly used alkylation agent is iodoacetamide (IAA), although it is less reactive than NEM and requires higher pH for efficient thiol alkylation. This elevated pH increases the risk of thioester hydrolysis, potentially compromising the preservation of *S*-acylation.^10,38–40^

During the capping step, tris(2-carboxyethyl)phosphine (TCEP) is often included to reduce disulfide bonds, minimizing false positives caused by disulfide exchange with uncapped cysteines during sample preparation. A sequential capping strategy using NEM followed by disulfide-forming reagent DTDP (2,2′-dithiodipyridine), termed “low-background ABE” (LB-ABE), has proven particularly effective. This approach achieves efficient thiol alkylation while minimizing non-specific background. Using LB-ABE, Zhou *et al.* identified 2895 putative long-chain *S*-acylated proteins in LNCaP cells, demonstrating its sensitivity and efficiency.^41^ This method has since been applied to study the role of cisplatin resistance in bladder cancer, and to analyze *S*-acylation in extracellular vesicles.^42,43^

#### 2.1.2 Enrichment strategies in ABE workflows for identifying long-chain *S*-acylated proteins

Enrichment of cysteines exposed by selective thioester hydrolysis is a critical step in identifying *S*-acylated proteins. Most studies use HPDP-biotin to label thiols released by hydroxylamine treatment under neutral pH conditions (Fig. 2B).^44–52^ However, alternative strategies have been explored to improve efficiency, sensitivity, and adaptability, including biotin-conjugated iodoacetamide (ICAT) and maleimide (BMCC-biotin), which also react selectively with free thiols.^29,53^

In 2011, Forrester *et al.* introduced the acyl resin-assisted capture technique, which streamlines *S*-acylation analysis by first blocking thiols followed by hydroxylamine-mediated cleavage, and then capturing and enriching thiols in a single solid-phase capture step using thiopropyl sepharose resin (Fig. 2A, step 1a).^54^ Widely adopted in both cell and tissue studies, acyl-RAC has become a staple in *S*-acylation profiling.^55,56^ A 2017 comparison with ABE revealed partial overlap in identified proteins, 241 by ABE, 144 by acyl-RAC, and 61 shared, highlighting differences likely due to experimental variability, enrichment efficiencies or variations in the reactivity and steric accessibility of cysteines, despite similar underlying chemical principles of the two methods.^57^

Alternative thiol-reactive solid supports, such as dithiodipyridine-functionalized magnetic nanoparticles and phenylmercury resin, have also been explored.^58,59^ More recently, Forrester *et al.* introduced a modified suspension trap that utilizes a thiol-reactive quartz to enable buffer exchange and hydroxylamine-mediated *S*-acyl enrichment.^60^

These studies taken together highlight the need to consider experimental design and the choice of enrichment strategy when interpreting results from studies of *S*-acylation. Tailoring enrichment methods to specific experimental requirements can maximize sensitivity and reliability in identifying *S*-acylated proteins.

#### 2.1.3 Advances in site-specific analysis of long-chain *S*-acylation

In 2010, Yang *et al.* pioneered site-specific *S*-acylation analysis by using HPDP-biotin-mediated peptide enrichment on streptavidin beads.^61^ This was followed by TCEP-mediated release of free cysteine-containing peptides, and subsequent analysis by LC-MS/MS.

Forrester *et al.* expanded on this by integrating acyl-RAC with site-specific *S*-acyl exchange: MMTS-blocked proteins were captured on thiopropyl sepharose, digested on bead, and *S*-acylated peptides were released with dithiothreitol (DTT).^54^ This integrated approach streamlined the identification of *S*-acylation sites by combining enrichment and digestion into a unified workflow. A similar strategy was reported by Gould *et al.* in 2015, who substituted thiopropyl sepharose with phenylmercury resin to capture *S*-acylated peptides, thus providing researchers with an alternative solid-phase support for peptide enrichment.^59^

In 2017, Collins *et al.* achieved a significant breakthrough, identifying 906 *S*-acylation sites across 641 proteins in the mouse brain proteome, representing the most extensive dataset of its kind at the time.^62^ A key innovation in their workflow was the use of molecular weight cutoff filters to efficiently remove small molecule reagents, replacing the precipitation methods commonly used in previous studies while improving peptide recovery. Zareba-Koziol *et al.* developed PANIMoni, a method which enables simultaneous site-specific detection of *S*-nitrosylation and *S*-acylation in a single experimental workflow.^63^ In a chronic stress model in murine brains, 813 *S*-acylated and 620 *S*-nitrosylated cysteine sites were identified on 465 and 360 proteins, respectively, with PTM crosstalk observed in 122 proteins including receptors, scaffolding proteins, regulatory proteins and cytoskeletal components. Later, Forrester *et al.* applied their acyl-trap strategy with site-specific resolution as well.^60^ Their method, utilizing thiol-reactive quartz, was compatible with both protein level as well as site-specific *S*-acylation detection. When combined with isobaric labeling, it achieved high sensitivity with as little as 20 μg of input material, a substantial reduction from the typical >500 μg requirement. This advancement opens new possibilities for *S*-acylation profiling in low-input samples such as plasma and secretomes.

These methodological innovations underscore a growing trend in the field toward site-specific analysis of *S*-acylation, which offers enhanced resolution and functional insights. As accumulating evidence reveals that individual proteins can harbor multiple *S*-acylation sites, potentially modified at varying stoichiometries and linked to distinct functional states, site-specific analysis will be essential for disentangling the complexity of these *S*-acyl-proteoforms and understanding their biological significance.^64,65^ Looking ahead, the integration of multiple proteases and the development of more sensitive analytical workflows hold promise for expanding the detectable *S*-acylation landscape and uncovering previously inaccessible modification sites.

#### 2.1.4 Pros, cons, and considerations in the use of acyl-biotin exchange-related strategies

A major challenge of the acyl-biotin exchange workflow is the complete capping of free cysteines, which is essential to minimizing background and ensure the specificity of the assay. Incomplete capping can lead to non-specific labeling, complicating the identification of true *S*-acylated proteins/peptides. To control for this, samples lacking hydroxylamine treatment are typically included to distinguish genuine *S*-acylated proteins/peptides from background.

The fold-change in protein abundance between +HA and −HA conditions provides a useful confidence metric, with higher fold enrichment in +HA samples suggesting *bona fide S*-acylation. Accurate quantification is critical for this analysis, as fold-change thresholds for defining *S*-acylated proteins can vary across studies. Researchers should carefully consider the criteria used to classify *S*-acylated proteins, as overly stringent or lenient cutoffs may skew results.

It is important to note that both acyl-RAC and ABE are not exclusively specific to long-chain *S*-acylation; they capture many proteins containing a thioester linkage, including catalytic intermediates such as those formed by ubiquitin-conjugating enzymes. Therefore, data must be critically evaluated to account for potential false positives.

Lastly, the SwissPalm database is a useful reference for putative *S*-acylated proteins; however, its aggregated nature means that caution is needed when interpreting entries, as study-specific variabilities and accumulated false discovery rates may inflate confidence.^1^ Ultimately, careful experimental design, rigorous controls, critical data interpretation, and thoughtful validation against databases like SwissPalm are essential for reliable ABE-based profiling of the *S*-acyl-proteome.

### 2.2 Lipid metabolic labeling for identifying long-chain *S*-acylated proteins

Traditional lipid-centric methods for detecting *S*-acylated proteins relied on the metabolic incorporation of radiolabeled fatty acids into proteins, followed by immunoprecipitation.^66,67^ While these approaches were valuable, they had notable limitations, including prolonged exposure times for autoradiography, limited sensitivity, and health concerns associated with handling radioactive materials.

**Fig. 3:**
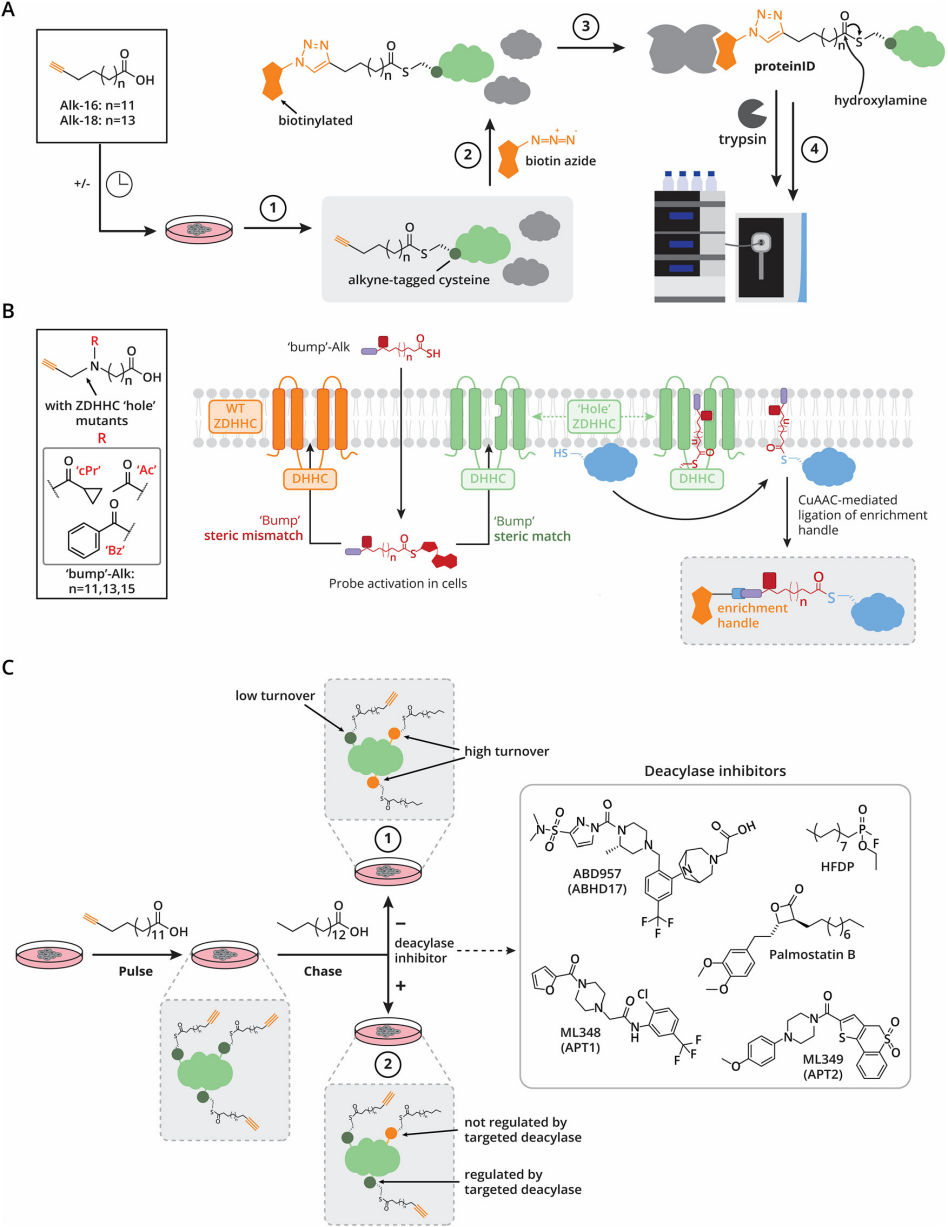
Lipid metabolic labeling workflows to detect *S*-acylated proteins and sites. (A) Classical LML: step 1. Cells are treated with various clickable fatty acid analogues resulting in alkyne-tagged proteins. Step 2. Alkyne-tagged proteins are conjugated to a biotin-azide reagent. Step 3. Biotinylated proteins are enriched using NeutrAvidin resin. *S*-Acylated proteins/peptides are selectively eluted of the NeutrAvidin resin using hydroxylamine. Step 4. Proteins are digested using *e.g.* trypsin (if performing a siteID workflow, biotinylated proteins are digested prior to enrichment), subjected to peptide clean-up and subsequent LC-MS/MS measurement. (B) Schematic representation of the “bump-and-hole” strategy for selective ZDHHC substrate identification. Cells are labelled with clickable “bump”-Alk probes (bumps: cPr = cyclopropyl, Ac = acetyl, Bz = benzoyl). Following intracellular activation by acyl-CoA ligase, transfer of the “bump”-Alk probes to wild-type ZDHHCs is blocked by steric hindrance, whereas “hole”-engineered ZDHHCs accommodate the probes during auto-acylation. The modified ZDHHC enzyme subsequently transfers the “bump”-Alk probe to its substrates. After cell lysis, enrichment handles are installed selectively on substrates carrying the “bump”-Alk modification through CuAAC-mediated click chemistry, enabling downstream enrichment and analysis. Schematic representation was inspired by Ocasio *et al.*^80^ (C) Schematic of two typical pulse-chase experiments, showing a multi-*S*-acylated protein to highlight the need for site-specific analyses. Cells are first labelled with clickable fatty acid analogues (“pulse”), followed by a wash-out period with palmitic acid (“chase”). Step 1. The palmitic acid chase is compared to a vehicle chase to gain information on lipid turnover. Step 2. The palmitic acid chase is supplemented with a deacylase inhibitor or a vehicle. Sites with a high turnover rate will quickly replace their alkynyl-acyl group with palmitate, sites that have low turnover rate, or that are stabilized by acyl-protein thioesterase inhibition, will retain their alkynyl-acyl group during the chase.

To address these challenges, modern lipid metabolic labeling (LML) strategies employ clickable fatty acid analogues, such as ω-azido- or alkynyl-fatty acids (Fig. 3A). These probes are recognized and processed by the endogenous cellular lipid metabolic machinery. Once inside the cell, the fatty acids are converted into fatty acyl-CoA intermediates, loaded onto ZDHHC *S*-acyltransferases, and subsequently transferred to substrate proteins. Clickable fatty acid analogues with varying chain lengths and degrees of saturation have been explored.

After the invention of LML by Hang *et al.*,^68^ LML was integrated for the first time with mass spectrometry proteomics by the Berthiaume group, who used Az-C14-CoA to label proteins in rat liver mitochondrial matrices. This study identified 19 novel acylated proteins and confirmed two previously known acylated proteins.^69^

Later, Alk-C18 (17-octadecynoic acid or 17-ODYA) was employed by Martin *et al.* to profile *S*-acylated proteins in membrane fractions of Jurkat T cells, identifying 125 *S*-acylated proteins.^70^ Similarly, Yount *et al.* used Alk-C18 to study the acyl-proteome in the DC2.4 dendritic mouse cell line, identifying 157 acylated proteins.^71^ These pioneering studies demonstrated the utility of lipid metabolic labeling for identifying proteins modified by long-chain *S*-acylation.

Since then, lipid metabolic labelling has enabled the identification of hundreds of long-chain *S*-acylated proteins, highlighting their roles in diverse biological processes, including inflammation,^72,73^ cancer,^74^ and viral infections.^75^ However, a critical limitation of this approach is its inability to differentiate between thioester-linked acyl groups and those attached *via* amide or ester bonds. Li *et al.* addressed this by including hydroxylamine controls in lipid metabolic labeling experiments, enabling more specific identification of *S*-acylated proteins. Using a SILAC-based quantitative approach, they identified flotillin-2 as a substrate for ZDHHC5.^76^

#### 2.2.1 Differentiation between various lipids through lipid metabolic labeling

Lipid metabolic labelling enables precise control over fatty acid chain lengths, aiding the study of lipid incorporation efficiency and substrate specificity of specific lipids. Wilson *et al.* showed that fatty acid analogues of varying chain lengths (Alk-C14, -C16, and -C18, where the number denotes the total number of carbons) label proteins with varying efficiency, likely reflecting preferences of specific ZDHHC enzymes, which exhibit chain-length selectivity.^77,78^ Building on this, Nůsková *et al.* found Az-C15 and Az-C17 can label distinct protein subsets.^79^

Moreover, Pradhan *et al.* identified that saturated very long-chain fatty acids (C20-alkyne) modify mixed-lineage kinase-like protein (MLKL), a necroptosis regulator.^81^ Thinon *et al.* extended these findings by showing that unsaturated fatty acid analogues can also be incorporated into proteins, albeit less efficiently than saturated counterparts.^82^

A recent study directly detected alkyne-tagged fatty acids on peptides and observed that these fatty acids can be metabolically extended or shortened prior to incorporation into proteins – highlighting the need to consider lipid remodeling when interpreting metabolic labeling data.^83^

#### 2.2.2 Analysis of long-chain *S*-acylation dynamics through lipid metabolic labeling

One of the key advantages of lipid metabolic labeling over acyl-biotin exchange is its ability to provide precise temporal control over lipid incorporation into proteins. This unique feature enables pulse-chase experiments, which are instrumental in studying the dynamics of long-chain *S*-acylation (Fig. 3C). For instance, pulse-chase experiments using alkyne-tagged lipids followed by a chase with palmitic acid have revealed a subset of dynamically *S*-acylated proteins (Fig. 3C, workflow 1).^84^ Incorporating broad-spectrum palmitoyl-protein thioesterase inhibitors in these assays further revealed dynamically long-chain *S*-acylated proteins that are regulated by acyl-protein thioesterases (Fig. 3C, workflow 2).^84^ Time-resolved lipid metabolic labeling using Alk-C18 has uncovered kinetically distinct protein clusters with varying lipid turnover rates. Here, the inclusion of the broad-spectrum *S*-acyl thioesterase inhibitor HFDP significantly increased Alk-C18 incorporation, indicating a key role for thioesterase activity in modulating *S*-acylation dynamics.^39^

The pulse-chase principle has also been adapted to identify specific *S*-acyl-protein thioesterase substrates. For example, use of the ABHD17-selective inhibitor ABD957 enabled the identification of ABHD17 targets such as SCRIB, MPP6, NRAS, and GNA12.^85^ These studies underscore the utility of lipid metabolic labeling in unraveling the dynamic regulation of *S*-acylation and its role in cellular signaling and homeostasis.

Additionally, lipid metabolic labeling has been adapted for “bump-and-hole” strategies. This approach relies on structure-guided engineering of paired ZDHHC “hole” mutants and “bumped” chemically tagged fatty acid probes, which enable selective probe transfer to specific protein substrates with excellent discrimination over wild-type ZDHHCs (Fig. 3B). Bumped lipid probes are dually functionalized with a bulky substituent of variable shape and size (*e.g.*, acetyl, cyclopropyl, or benzoyl) together with an alkyne tag for enrichment or visualization (Fig. 3B, left). To achieve selective labeling, the probes must be complementary to the engineered ZDHHC, in which a “hole” is created in the lipid-binding pocket by mutating a bulky amino acid to a smaller residue. This exclusive transfer by the engineered ZDHHC facilitates identification of its specific substrates, thereby overcoming the challenges posed by ZDHHC redundancy and co-regulation.

When coupled with chemical proteomics, this strategy enables the detection of low-abundance ZDHHC substrates and low-stoichiometry *S*-acylation sites. By bypassing substrate redundancy among ZDHHCs, it simplifies the analysis of the *S*-acylation landscape and supports identification of substrates for individual ZDHHC isoforms. Furthermore, by varying the bump size and position in the lipid probe and generating compatible “hole” engineered ZDHHCs, the design of paired “hole” mutants and “bumped” fatty acid probes is, in principle, feasible for every ZDHHC. To date, “bump-and-hole” strategies have provided insights into the substrate specificity of ZDHHC3, ZDHHC7, ZDHHC15, and ZDHHC20.^80,86^ Ocasio *et al.* further highlighted the potential of this approach for drug discovery and target validation in ZDHHC-associated diseases, as well as for the development of novel, selective ZDHHC inhibitors.

## 3. Complementary use of acyl-biotin exchange and lipid metabolic labeling for profiling long-chain *S*-acylation

Acyl-biotin exchange and lipid metabolic labeling are widely used, complementary strategies for probing long-chain *S*-acylation. Each offers distinct advantages: ABE provides broad coverage of the long-chain *S*-acyl-proteome, while lipid metabolic labeling excels at capturing dynamic, high-turnover *S*-acylations. When used in tandem, these approaches can yield a more comprehensive view of the *S*-acyl-proteome and can identify a subset of high-confidence long-chain *S*-acylated proteins which are detected by both methods.

Jones *et al.* demonstrated the value of this dual approach in the parasite *Plasmodium falciparum*, revealing a partially overlapping yet distinct set of *S*-acylated proteins identified by each method. Their integration of ABE and lipid metabolic labeling led to the identification of 409 long-chain *S*-acylated proteins.^87^

In mammalian systems, Won and Martin compared acyl-resin-assisted capture with Alk-C18 metabolic labeling in HEK293-T cells. Acyl-RAC yielded a far larger dataset, 728 proteins *versus* 195 from lipid metabolic labelling, with only ∼10% overlap. This limited intersection raised intriguing possibilities: long-chain *S*-acylation may be more stable than previously thought, or native palmitate might outcompete Alk-C18 for incorporation.^39^

Building on these insights, Thinon *et al.* developed an integrated protocol combining LML with hydroxylamine cleavage, allowing precise site-specific identification of long-chain *S*-acylation sites. In their work on profiling *S*-acylation sites on IFITM3, they highlighted the limitations of applying ABE alone, which can capture all thioester-linked cysteines regardless of lipid-context, potentially inflating false positives. As such, they advocated for parallel application of both methods to enhance specificity and accuracy.^56^

More recently, Sardana *et al.* applied the dual approach to investigate the role of long-chain *S*-acylation during retinoic acid-induced differentiation of SH-SY5Y cells into a neuronal phenotype. Their integrated strategy uncovered 2002 *S*-acylated proteins, with 606 identified by both ABE and lipid metabolic labeling. Many of these proteins were critical for neuronal differentiation, underscoring the regulatory potential of long-chain *S*-acylation in key cellular processes such as neuronal differentiation.^28^

Collectively, these studies illustrate the power of combining ABE and lipid metabolic labeling to chart the landscape of long-chain *S*-acylation. By harnessing their complementary strengths, researchers can gain deeper insights into the dynamics, specificity, and functional significance of this lipid modification across diverse biological systems

## 4. Direct detection of protein long-chain *S*-acylation

While the indirect strategies are invaluable for studying protein *S*-acylation, direct analytical approaches are highly sought after. Direct methods offer critical advantages, including identification of the attached lipid species, reduced susceptibility to false positives, isoform-specific resolution, and stoichiometric insights. As a result, multiple studies have focused on directly detecting *S*-acylation at the peptide and protein levels.

### 4.1 Peptide-centric (bottom-up) approaches

**Fig. 4:**
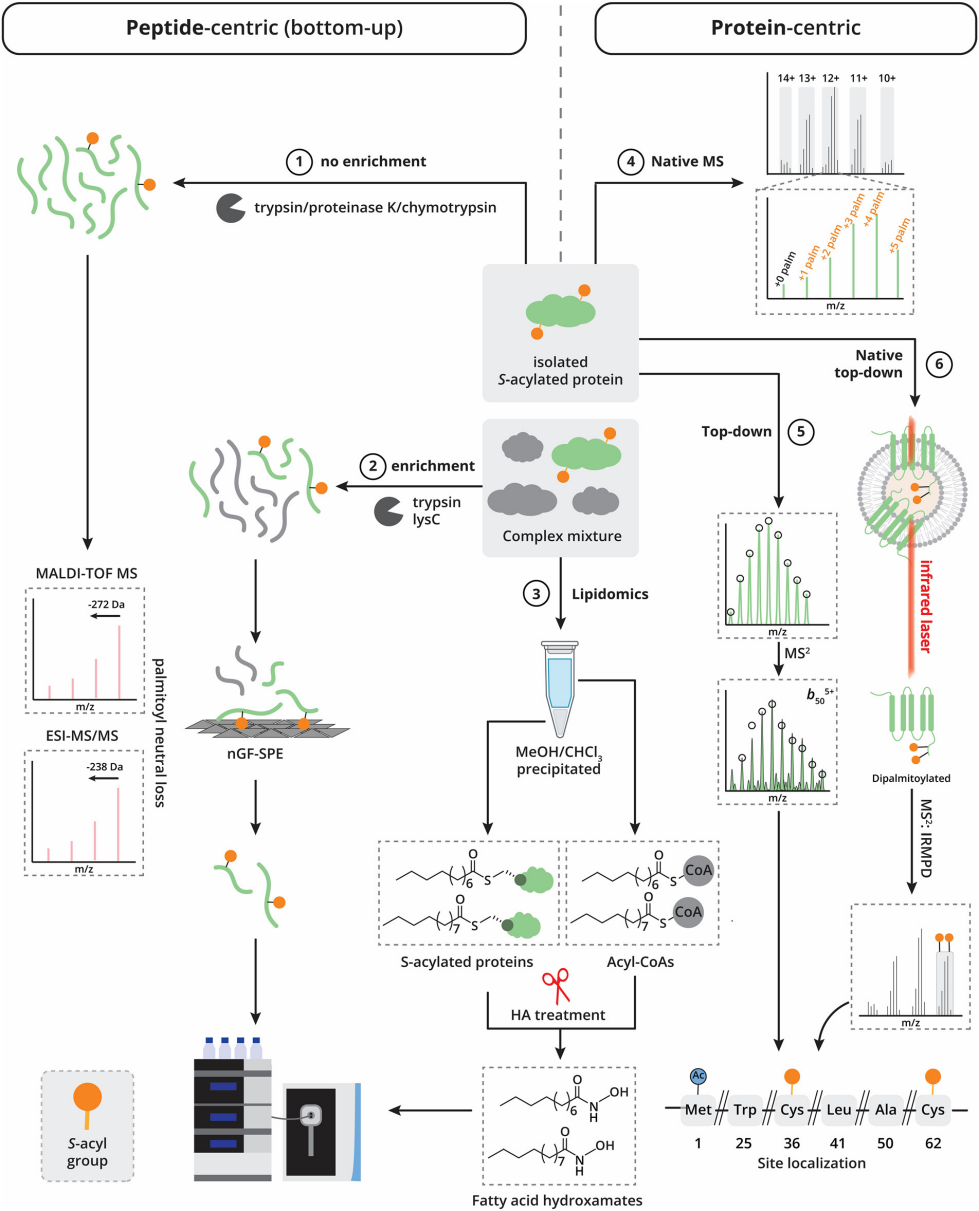
Current techniques in the direct detection of protein *S*-acylation with mass spectrometry. Peptide-centric (bottom-up) approaches: workflow 1. Isolated proteins are digested with protease(s) and separated (off-line or on-line) by liquid chromatography using a C_4_, C_8_ or C_18_ stationary phase (off-line for MALDI-TOF-MS, on-line for ESI-MS/MS) and analyzed either by MALDI-TOF MS or ESI-MS/MS. Workflow 2. Complex mixtures are digested and *S*-acylated peptides are enriched using nGF-SPE followed by LC-MS/MS analysis. Lipidomics: workflow 3. Complex mixtures undergo multiple precipitations, after which the pellet and MeOH layer are treated with hydroxylamine to acquire fatty acid hydroxamates from *S*-acylated proteins and acyl-CoAs, respectively. Protein-centric approaches: workflow 4. Native/intact MS approaches analyze proteins without digestion or fragmentation. These approaches can distinguish between different *S*-acyl-proteoforms but cannot pinpoint individual *S*-acylation sites. Workflow 5. Top-down MS can identify distinct *S*-acylation sites after fragmentation of the protein backbone. Workflow 6. Native top-down MS ejects complexes from their native membrane environment using a CO_2_ laser. IRMPD fragmentation allows for protein sequencing with the advantage that *S*-acylations are not lost during fragmentation.

In the 1990s, mass spectrometry techniques like matrix-assisted laser desorption/ionization time-of-flight mass spectrometry (MALDI-TOF-MS) and plasma desorption TOF-MS were used to ionize and study lipidated peptides, identifying lipidation events such as palmitoylation and myristoylation (Fig. 4, workflow 1). Studies by Steinert *et al.*, Hensel *et al.*, and others, discovered *S*-acylation on proteins like transglutaminase 1 (TGase 1), p12E, and surfactant-associated protein C (SP-C), some of which proved to be crucial for membrane association.^88–92^ In addition MALDI-TOF MS was used to identify *S*-acylation of hemagglutinin of influenza viruses.^93,94^ These foundational studies paved the way for current direct analysis techniques by highlighting the power of direct MS analysis to uncover previously uncharacterized sites of lipid modifications.

In the past decade, the field has largely shifted from MALDI-TOF-MS to LC-ESI-MS/MS, with only a few notable exceptions. For instance, Montigny *et al.* used MALDI-TOF-MS to explore lipidation diversity on a single *S*-acylation-susceptible cysteine in rabbit sarcolipin (SLN), a 31-residue membrane protein. They identified either palmitic or oleic acid as *S*-acyl groups through comparative peptide analysis before and after hydroxylamine treatment. Building on this approach, Nůsková *et al.* further demonstrated that *S*-acylation of GNAI at Cys3 by C16:0 or C18:1 yields divergent outcomes, C16:0 promotes localization to detergent-resistant membrane, while C18:1 does not. This shows that specific long-chain fatty acids may have distinct regulatory effects.^95,96^

Further work by Hoffman and Kast revealed characteristic neutral losses associated with *S*-palmitoylation, 238 Da (C_16_H_30_O) in ESI and 272 Da (C_16_H_32_OS) in MALDI-TOF, although diagnostic ions for *S*-palmitoylation remained elusive (Fig. 4, workflow 1).^97^

To evaluate the stability of *S*-acylation under different fragmentation conditions, Ji *et al.* analyzed the fragmentation behavior of synthetic *S*-palmitoylated peptides under multiple fragmentation techniques. Among collision-induced dissociation (CID), higher-energy collision dissociation (HCD), electron-transfer dissociation (ETD), and electron-capture dissociation (ECD), ETD was most effective, preserving the labile palmitoyl group while enabling robust backbone fragmentation.^25,98^

In parallel with advances in fragmentation strategies, various liquid chromatography techniques have also been employed to directly analyze *S*-acylated peptides. Thinon *et al.* applied C_18_ LC-ESI-MS/MS after chymotrypsin digestion to identify long-chain *S*-acylation on IFITM3, with palmitate as the predominant lipid on Cys71, Cys72 and Cys105.^56^ Minor oleate and palmitoleate modifications were also detected, reflecting the complexity of cellular lipid pools. Similarly, Gottlieb *et al.* employed LC-ESI-MS/MS after C_4_ chromatography to detect *S*-palmitoylation events on the cysteine-rich domain of DHHC3.^15^

Wang *et al.* further demonstrated the utility of C_4_ and C_8_ chromatography, coupled with ESI, to validate *S*-palmitoylation on lens proteins MP20 and AQP5. Using iodoacetamide-palmitate exchange, they quantified sub-stoichiometric palmitoylation levels on AQP5 in different lens regions, implicating *S*-acylation in the spatial regulation of protein translocation during lens fiber cell differentiation.^48^

Most recently, Ji *et al.* developed nano-graphite fluoride solid-phase extraction (nGF-SPE), a proteome-wide enrichment strategy for *S*-acylated peptides (Fig. 4 workflow 2). This approach enabled identification of 1119 lipid-modified peptides, including those bearing palmitate, palmitoleate, myristate, and octanoate, and delivered a 100-fold sensitivity increase compared to direct protein lysate analysis. However, despite these improvements, *S*-acylated peptides comprised only 1.7% of the total identified peptide pool, indicating a further refinement is needed for robust enrichment strategies for large-scale *S*-acyl-proteomics.^99,100^

### 4.2 Protein-centric approaches

The analysis of *S*-acylation on intact proteins offers significant advantages over peptide-based approaches, as it avoids the challenges associated with hydrophobic peptide handling. By analyzing the intact protein, the hydrophilic regions can compensate for the high hydrophobicity often present near *S*-acylation sites. This method also facilitates the determination of *S*-acylation stoichiometry.

#### 4.2.1 Intact protein analysis

In native or intact protein analysis the mass of the complete protein is analyzed without protein digestion or fragmentation. Rodenburg *et al.* utilized native mass spectrometry to analyze claudins 3, 4, and 6 (Cld3, Cld4, Cld6), a group of transmembrane proteins that are generally difficult to ionize (Fig. 4, workflow 4).^101^ In their analysis, they observed peaks corresponding to the theoretical masses of the unmodified protein sequences, alongside five additional peaks for Cld3. Each subsequent peak reflected a relative mass increase of 238 Da, consistent with the addition of palmitoyl chains. These findings indicated that Cld3 can be modified with up to five palmitates, with the most prevalent isoform containing four palmitoyl chains, suggesting the presence of four dominant *S*-acylation sites. Alanine scanning mutagenesis confirmed the four key *S*-acylation sites. Furthermore, introducing non-native *S*-acylation sites into Cld3 demonstrated that membrane–protein function imposes evolutionary constraints on native palmitoylation sites. These results support a model where membrane–protein palmitoylation is largely stochastic, governed by the accessibility of ZDHHC's to cysteines on membrane-embedded proteins rather than a strict substrate-sequence motif.

Building on this work, the same group later applied the native mass spectrometry strategy to analyze CD9 and CD81.^102^ Their findings revealed that these tetraspanins undergo non-stochastic *S*-acylation by both palmitic and stearic acids. Notably, the lipidation of these proteins influenced their interaction partners within tetraspanin clusters without altering the number of molecules in the clusters. This highlights the nuanced role of *S*-acylation in modulating protein–protein interactions and membrane organization.

#### 4.2.2 Top-down proteomics

Top-down mass spectrometry-based proteomics has emerged as a powerful tool for analyzing intact proteins and their post-translational modifications. Unlike native MS, top-down MS provides the added advantage of directly identifying *S*-acylation sites on proteins by fragmentation of the protein backbone in the mass spectrometer to retrieve protein sequence information, enabling a more detailed investigation of the interplay between different PTMs (Fig. 4, workflow 5).

Rogers *et al.* used a photocleavable surfactant (Azo) to solubilize membrane proteins, a key challenge in top-down MS workflows.^103^ This innovative approach facilitated the analysis of membrane protein samples using online reversed-phase LC-MS/MS coupled with ultrahigh-resolution Fourier-transform ion cyclotron resonance (FTICR) MS. By leveraging these strategies, the researchers successfully studied proteoforms of the phospholamban (PLN) protein, revealing critical insights into the crosstalk between long-chain *S*-acylation and phosphorylation.

In a 2025 study, Lutomski *et al.* introduced the use of native top-down MS to directly release membrane proteins, including the G protein-coupled receptor (GPCR) Rhodopsin and its interacting partners, from their native lipid bilayer *via* infrared laser irradiation (Fig. 4, workflow 6).^65^ This method enabled the resolution of multiple rhodopsin proteoforms and facilitated top-down protein sequencing using infrared multiple photon dissociation (IRMPD) (Fig. 4, workflow 6). Notably, the authors characterized a complex PTM landscape, identifying two palmitoylated cysteines on rhodopsin (C322 and C323), along with farnesylation and geranyl-geranylation on phosphodiesterase 6 (PDE6) and several G proteins. Remarkably, despite their reputed lability, the palmitoylated cysteines were preserved during IRMPD-induced fragmentation, which still produced extensive backbone cleavage, enabling PTM localization (Fig. 4, workflow 6). This capability, not previously demonstrated in a top-down set-up, opens new avenues for drug discovery by enabling direct assessment of how long-chain *S*-acylation influences drug binding and mediates protein–protein interactions. These findings underscore the utility of (native) top-down MS for elucidating the functional interplay between PTMs, identifying drug–proteoform interactions and open new avenues for understanding membrane protein regulation.

Complementing protein-focused approaches, recent developments in protein-centric lipidomics have further expanded the toolbox for probing long-chain *S*-acylation. Busquets-Hernandez *et al.* applied a method to quantify fatty acid hydroxamates (FAHs) released from both protein-bound and acyl-CoA fractions following hydroxylamine treatment (Fig. 4, workflow 3).^104^ Their approach captured the diversity of thioester-linked lipids and revealed distinct C18:0/C16:0 modification ratios in non-cancer (1.5 : 1) *versus* cancer cell lines (1 : 1), reflecting differential lipid demands. The study also uncovered marked differences between the *S*-acylome of cultured cells and tissues, with cultured cells exhibiting a higher proportion of mono-unsaturated fatty acid (MUFA) modifications, likely influenced by fetal bovine serum (FBS) in culture media. These findings emphasize the importance of considering culturing conditions when interpreting *S*-acyl-proteomic data.

## 5. Conclusions and outlook

In conclusion, advances in mass spectrometry-based tools have significantly deepened our understanding of protein *S*-acylation, with a clear shift towards site-specific workflows. These approaches are essential for resolving the functional complexity of multi-site *S*-acylated proteins and will benefit from enhanced sequence coverage through the incorporation of alternative proteases. The many different MS-based strategies to study long-chain *S*-acylation have their intrinsic strengths and weaknesses and often are complementary. An overview of the strengths and weaknesses of the individual MS based strategies can be found in Table 1. Complementary strategies such as LML and ABE/acyl-RAC should be employed in parallel to maximize sensitivity, and coverage of the *S*-acyl proteome.

**Table 1:** Comparative analysis of MS-based strategies to study protein long chain *S*-acylation

Strategy	Site-specific	Lipid-specific	Analysis in tissue	Analysis in cell culture	Sensitive to false positives	Analysis in complex proteomes	Stoichiometry analysis	Sensitivity	Throughput	Ref.
**Acyl-biotin exchange**										
Protein-centric	−	−	+	+	++	+	−	++	+	28
Peptide-centric	+	−	+	+	+	+	−	+	+	105
**Acyl-Rac**										
Protein-centric	−	−	+	+	++	+	−	++	+	57
Peptide-centric	+	−	+	+	+	+	−	+	+	60
**Lipid metabolic labeling**										
Protein-centric	−	−	−	+	+	+	−	++	±	28, 69 and 70
Peptide-centric	+	±	−	+	−	+	−	+	±	83
**Direct detection strategies**										
Bottom-up proteomics	+	+	+	+	−	+	−	−	+	100
Intact protein analysis	−	±	+	+	−	−	+	−	−	65, 101 and 102
Top-down proteomics	+	+	+	+	−	−	+	−	−	65 and 103

Importantly, emerging evidence indicates that protein *S*-acylation is more complex than the mere addition of palmitic acid to proteins. Busquets-Hernandez *et al.* reported that the attachment of C18 fatty acids to proteins *via* a thioester bond is the predominant modification in mouse brain, heart, kidney, liver, muscle, and white adipose tissue.^104,106^ Furthermore, Nůsková *et al.* demonstrated the functional importance of *S*-oleoylation of GNAI in potentiating EGFR signalling.^96^ As lipid substrate selectivity among ZDHHC acyltransferases becomes increasingly apparent, there is a critical need for methods capable of distinguishing lipid-specific *S*-acylation events. Although LML is well-suited for such studies, its most used formats result in the loss of the lipid moiety following hydroxylamine treatment. Moreover, fatty acid analogues can undergo metabolic processing prior to incorporation, complicating the interpretation of lipid-specific modifications. Techniques that preserve the lipid moiety, such as az-DADPS-biotin-based LML, offer a more reliable avenue for probing lipid heterogeneity.

The *S*-acylome is highly responsive to lipid composition in the culture environment, particularly exogenous sources such as fetal bovine serum. *In vitro* models may therefore not fully reflect physiological *S*-acylation profiles, and future studies should account for media composition when interpreting data. Furthermore, robust quantitative strategies will also be essential, particularly for dissecting the roles of regulatory enzymes (*e.g.* ZDHHCs, thioesterases) and for evaluating candidate therapeutics. Moreover, studying dynamic biological processes such as signaling, differentiation, and disease progression requires quantitative workflows capable of capturing temporal changes in *S*-acylation. As the field moves toward mechanistic insights and translational applications, precise quantification will become increasingly indispensable.

While the direct detection strategies have already provided highly valuable insights, much progress is needed for this aspect of the *S*-acylation profiling field to move forward. Peptide-level enrichment strategies still face limitations, including low enrichment efficiency and shallow depth of coverage. Optimization of these methods through improved chromatography, alternative solvents, and higher-efficiency capture strategies will be key to reaching the analytical maturity seen in other post-translational modifications, such as phosphorylation. Enhancing fragmentation efficiency for *S*-acylated peptides is another priority. While the palmitoyl group is typically labile, successful retention with ETD (peptide-level) and IRMPD (protein-level) suggests that specialized fragmentation methods may improve identification rates.

Finally, (native) top-down mass spectrometry continues to be hindered by low sequence coverage and challenges in PTM localization due to data complexity. Improved computational tools for deconvolution and PTM assignment will be essential to fully unlock the potential of top-down approaches for *S*-acylation profiling.

## Author contributions

A. E. N., S. S., and M. P. B. conceptualization; A. E. N. and M. P. B. writing – original draft; M. P. B. supervision; A. E. N. and S. S. visualization; A. E. N., S. S. and M. P. B. writing – review and editing; M. P. B. funding acquisition.

## Conflicts of interest

There are no conflicts to declare.

## Data Availability

No primary research results, software or code have been included and no new data were generated or analyzed as part of this review. Figures were made using ChemDraw 19.1, Biorender.com and Adobe Illustrator (2025).
